# Thermo-Mechanical Analysis in the Fresh Fruit Cold Chain: A Review on Recent Advances

**DOI:** 10.3390/foods10061357

**Published:** 2021-06-11

**Authors:** Alemayehu Ambaw, Tobi Fadiji, Umezuruike Linus Opara

**Affiliations:** 1 SARChI Postharvest Technology Research Laboratory, Africa Institute for Postharvest Technology, Faculty of AgriSciences, Stellenbosch University, Stellenbosch 7602, South Africa; tsige@sun.ac.za (A.A.); fadiji@sun.ac.za (T.F.); 2 UNESCO International Centre for Biotechnology, Nsukka 410001, Enugu State, Nigeria

**Keywords:** simulation, modeling, computational fluid dynamics, computational structural dynamics, postharvest, cold chain, food loss and waste

## Abstract

In agro-food research and industry, mathematical models are being used to develop and optimize preharvest and postharvest operations, and their use has grown exponentially over the last decade. Generally, transport phenomena (such as airflow, heat, and mass transfer) during the cooling of horticultural products are complex; therefore, the use of computational modeling techniques is a valid alternative to expensive and difficult experiments because computers continuously become more powerful and less expensive, the software is readily available, and once a model is validated, it is a versatile tool to evaluate the effects of the operating and design parameters involved. In this review, thermo-mechanical modeling studies during postharvest handling are overviewed regarding the experimental, analytical, and computational approaches. The airflow, cooling kinetics, cooling uniformity, and the material and mechanical safety behavior of fresh fruit packaging boxes will be analyzed. Current concerns, challenges, and opportunities are discussed.

## 1. Introduction

Mathematical modeling complements testing and experimentation by reducing the total effort and cost of data acquisition in the agricultural sector. Mathematical models help to explain systems and quantify the effects of various factors on the performances of preharvest and postharvest handling processes. This approach stimulates interdisciplinary research on the topic. Most of the studies in this area were published in the journal category of horticulture and food science technology. 

In the preharvest period, mathematical models of 3D tree architectures are used in the development and optimization of automated field operations including automated pruning, automated tree branch training, and orchard spray applications [1,2,3]. A diverse range of mathematical models exists to describe the 3D geometric structure of plants. Traditionally, tree structure modeling methods such as the medial axis thinning algorithm [4,5] and stripe programming [2] were used to compute tree skeletons from a gradient intensity map. Recently, artificial-intelligence (AI)-based imaging and image analysis tools are making dramatic breakthroughs in classifications and object detection applications [6]. In another area, computational fluid dynamics (CFD) models of airflow and particulate transport within plant canopies were applied to analyze and optimize pesticide and fungicide spray application to agricultural fields and to assess the accompanying environmental contamination [3].

During harvest, models are used to develop and optimize picking robots. For instance, “stripe programming” was used to reconstruct the 3D structure of Chinese hickory trees, followed by the finite element method (FEM) to optimize the excitation force-frequency and amplitude that is efficient for harvesting [2]. The current trend in this area is to support the various automated field operations with deep-learning-based artificial intelligence (AI) technology for fruit and tree branch detection [2,6]. 

Geometric parameters (shape, size (major and minor diameters), volume) and physical properties (texture, firmness, color, density) of agricultural products are widely used to model crop growth and variations in physicochemical characteristics [7,8]. Particularly, fruit diameter is considered a very important index of fruit growth dynamics that can be monitored non-destructively during the growing season. To this end, it is frequently used to forecast harvest size [8].

During the postharvest period, mathematical models are used to study mechanical damages such as bruising from postharvest handling and road transport operations [9,10,11]. The thermo-mechanical analysis of packaging and cold chain management logistics has been assessed using various types of mathematical models [12,13,14,15]. Biophysical phenomena taking place during precooling [12,13,14], cold storage [15], refrigerated transport [16,17], retail (display) cooling systems [18,19] have been studied using mathematical models.

In this paper, progress made in the application of mathematical models in the postharvest period is reviewed. This paper consolidates and relates the various numerical modeling studies, specifically CFD and computational structural dynamics (CSD) in the last 10 years. The objectives, basic procedures, and implementation of the modeling approaches are reviewed. Advances achieved and current trends are discussed. This paper is expected to serve as a basis for further contributions in the area. 

## 2. Numerical Methods in the Produce Cold Chain Management

### 2.1. Effect of Loading Conditions on Stacked Fibreboard Cartons in the Distribution Chain 

**Figure 1:**
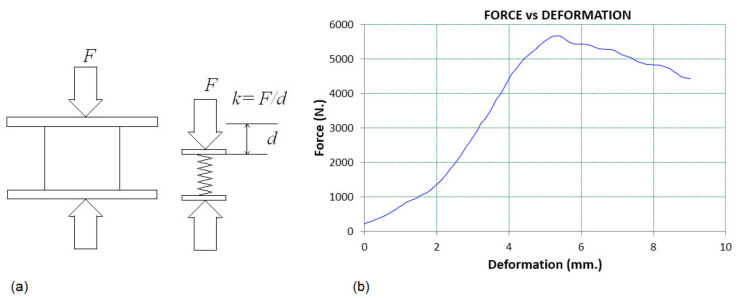
The force diagram of box compression test (**a**) and a typical box compression test graph (**b**).

The packaging of fresh fruit is a means of ensuring the delivery of produce from grower to consumer in a safe and convenient manner, at a minimum overall cost [12,13,14]. Because of their low cost and versatility, corrugated fiberboard cartons (CFCs) are the dominant and economical material for making shipping containers that are widely used for the distribution, transportation, and storage of produce. A significant challenge that affects the use of CFCs is the ability to maintain the mechanical strength of the cartons under cold chain conditions over a long period of time [20,21,22,23,24]. During storage and transportation, various types of forces subject the faces and corners of the carton to compression, bending, or tensile loading [22,24]. In pallet stacking, cartons are placed on top of one another, up to approximately 2 m in height. This subjects the bottom carton to carrying the load above, usually about 1000 kg. Hence, testing the box’s mechanical strength is crucial to making the packaging safe throughout the supply chain (Figure 1). Commonly, a box compression test (BCT) quantifies the compression strength of CFCs [20]. This test is a pure top-to-bottom compression load applied on a sample placed between two flat, parallel plates (Figure 1a). When the test begins, the upper plate moves downward slowly to exert load onto the specimen carton and the compressive force, *F* [N], versus the displacement, *d* [m], in a load-deformation curve (Figure 1b), obtained for analysis. The strength requirement in the distribution chain is established based on the total weight supported by the bottommost box in the stack of packages [20,21,22,23,24]. The load may be exposed to fluctuations in temperature and humidity, as well as other factors that affect performance such as excessive handling, pallet patterns, pallet deck board spacing, and box overhang. These unpredicted loads are accounted for by a factor of safety that is dependent on the supply chain [24,25]. For fresh fruit packaging with ventilation holes, several factors have been reported to influence its strength including the location, sizes, and shapes of the ventilation holes [20,21,22,23,24,25]. The ISO 12,048 and ASTM D642 are the commonly used standards for evaluating BCT.

Designing fresh fruit packaging boxes involves both lab tests and field tests in various configurations. Repeated tests on physical prototypes are crucial to evaluate the safety of new CFCs in the competitive world market. This requires repeated die cutter adjustments, which are time consuming and expensive [26]. Therefore, reliable analysis tools for the prediction of both the structural integrity and its interaction in the cold chain of new package designs are very important. As a novel technique, the mechanical analysis of individual CFCs and stacked and palletized CFCs has been assessed using computational structural dynamics (CSD) models [22,23,24]. This involves discretizing a large domain into many small elements, developing element equations, assembling the element equations for the whole domain, and solving the assembled equations to simulate and predict the mechanical responses in CFCs upon loading [27,28]. This technique uses a complex system of points called nodes, which make a grid called a mesh. This mesh is programmed to contain the material properties that define how the migration will occur. Although FEM started out as a mathematical technique, most FE analyses are now run through commercial software such as ABAQUS, MSC, ANSYS, and COMSOL, etc. FEM consists of three principal operations: pre-processing, analysis, and post-processing, which have been comprehensively discussed in the review by Fadiji et al. [29]. The pre-processing stage includes simplifying and modeling the geometry, selecting appropriate element types, and defining material properties including applying loads, boundary conditions, and constraints [27,28,29,30]. The analysis step involves solving the set of mathematical equations that describe the behavior of each element.

### 2.2. The Influence of Vent Design of Fibreboard Cartons on Produce Precooling Management

Temperature is the most important factor that influences the quality of harvested horticultural crops. The internal flesh (core) temperature of the fruit determines the rate of respiration and other enzymatic reactions. Internal temperature also determines respiration heat generation [31]. Temperature interacts with other internal and external factors and affects spore germination and pathogen growth [31,32]. The type of produce, maturity, presence of injuries, etc., are important factors that determine the effect of temperature. For most perishable horticultural commodities, a temperature near 0 °C is optimal. Temperature control is vital, and cooling must start as quickly as possible after harvest to sufficiently slow the respiration process for a longer fruit shelf life. As a rough guide, an hour delay in cooling reduces a product’s shelf life by one day [33]. Hence, the rapid cooling of fruits and vegetables immediately after harvest is crucial for the effectiveness of the cold chain.

**Figure 2:**
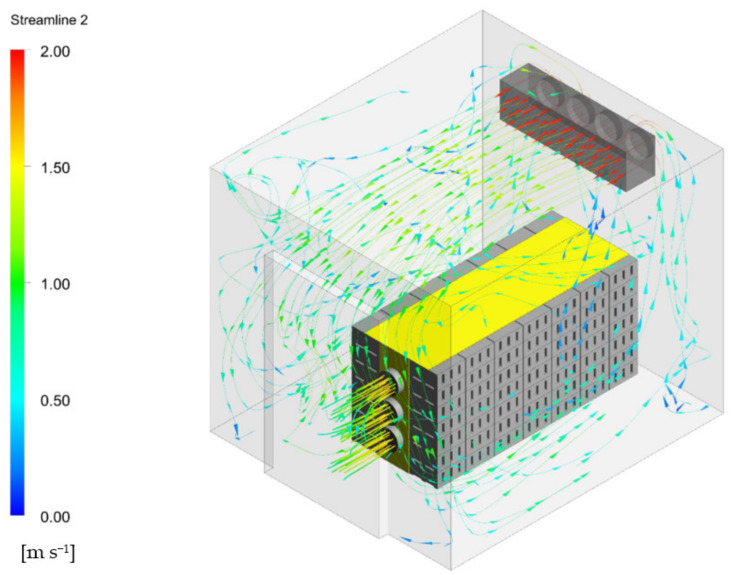
Configuration of precooling tunnel.

**Figure 3:**
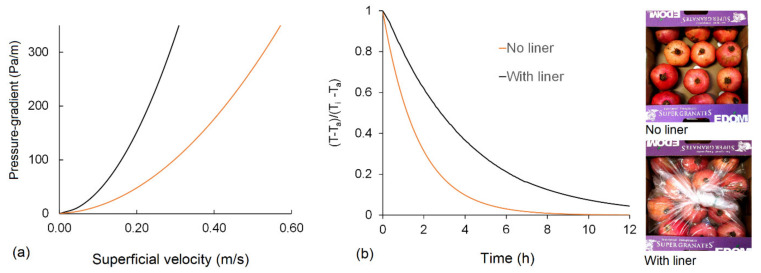
Measuring the effect of internal liner on the airflow resistance (**a**) and the accompanying cooling rate (**b**). Measurements taken for a pallet of packed pomegranate fruit. The packages without liner (No liner) and with liner wrapping (With liner) are shown on the right.

Rapid cooling is commonly accomplished by a forced air-cooling (FAC) system placed in refrigerated rooms. Fans force the cold air of the room through the stack of produce. Forcing the cold air through the stack increases the cooling rate significantly, allowing produce to be cooled much faster compared to room cooling [33,34]. The tunnel cooler is the most common FAC configuration (Figure 2). In this arrangement, two rows of stacked and palletized produce are placed on either side of an air return channel. A tarpaulin sheet or cover is placed over the pallets and the channel, and a fan draws air from the channel, directing the chilled air of the cold room through the produce [35]. Cooling times range from 1 h for cut flowers to more than 6 h for larger fruit [36]. The presence of additional packaging materials (such as bags, plastic films, paper wraps, etc.) increases the airflow resistance and hence the cooling time (Figure 3). Hence, package design should aim to enhance the flow rate and distribution uniformity of the cooling air during a precooling process.

The ease and uniformity of the cooling air distribution are significantly affected by the vent hole design (shape, position, and proportion) of the packaging box and package arrangement. Mathematical modeling methods, mainly computational fluid dynamics (CFD), have been successfully used to analyze the cooling rate and cooling uniformity of produce cooling processes [12,13,14]. Packaging design also affects the energy efficiency and carbon footprint of precooling operations [12,13,14,37]. For instance, the comparison of several pomegranate packaging designs showed significantly different cooling uniformity, cooling rate, and energy usage in a precooling process [13,37]. 

### 2.3. Cold Storage Room

**Figure 4:**
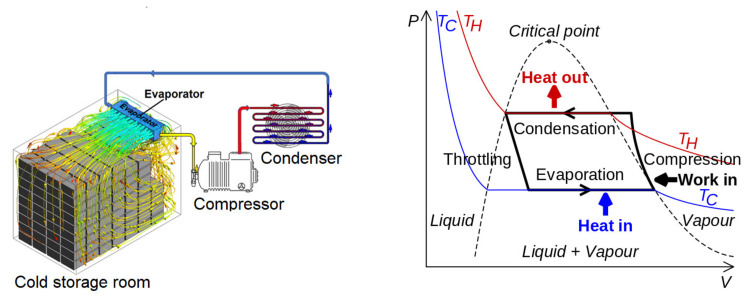
Illustration of a typical vapor compression refrigeration-based cold storage room (**left**) and the corresponding pressure–volume diagram of the refrigeration cycle (**right**).

Once precooled fruits are placed in a cold storage room, the temperature, humidity, and gas (O_2_, CO_2_, and ethylene) concentrations should be controlled at the optimum condition to keep produce fresh for longer. The uniform distribution of moisture and gas in the cold room atmosphere is crucial for efficient quality preservation. Fresh fruit cold storage rooms employ the vapor compression method of mechanical refrigeration [38]. The system basically has four components: a compressor, a condenser, a thermal expansion valve (also called a throttle valve or metering device), and an evaporator. Figure 4 illustrates a typical cold storage room with a wall-mounted evaporator unit and compressor, condenser, and valve units placed outside the storage area. The design and operation of a cold storage room should consider the heat load factors, such as solar radiation on the walls and ceiling (especially when a cold room is not accommodated in a bigger building), the infiltration of air by frequent door openings, the heat of respiration from stored produce, cooler fan load, light load, and other miscellaneous loads [15,39]. Additionally, the flow resistance induced by the packages is important parameters that affect the air, moisture, temperature, and gas distribution. Uneven distribution of cooling air, humidity, and gas in the room can cause non-uniform produce quality and safety. Hence, understanding the type and design of packages—e.g., intermediate bulk bin, or small corrugated fiberboard shipping boxes, the nature and type of internal packaging materials, etc.—is crucial [15,38,39].

### 2.4. Reefer Container

**Figure 5:**
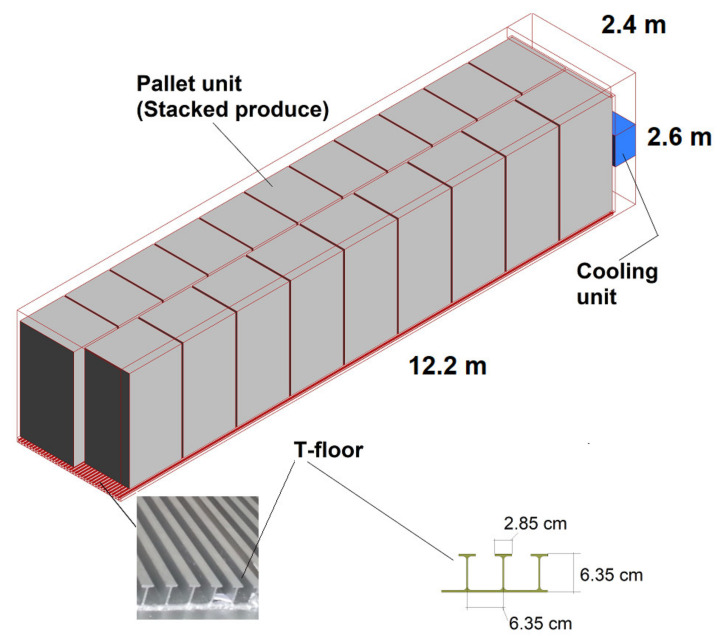
Schematic depiction of bottom-air-delivery T-bar reefer and a closer view of the T-bar floor structure.

Fruits and vegetables for a distant market should be kept cool and at optimum conditions during transit. This is accomplished in a refrigerated transport container [16,17,39] that has its own self-powered refrigeration unit. However, the refrigeration capacity of a reefer container is limited, and it is only possible to keep the produce at the optimum temperature, not to meet the precooling requirement. Thermal testing of refrigerated equipment is a vital activity to benchmark the thermal performances and ultimately maintain the quality of products being transported. For refrigerated containers used for sea, rail, and road transport throughout the world, compliance with the ISO standard 1496-2 must be attained [40]. The three key thermal tests considered in the standard are: insulation effectiveness, air tightness, and the refrigeration capacity of the unit. Like the precooling and cold storage units, package design (size of boxes and vent hole design) and arrangement (stacking patterns) play crucial roles in determining the performance of energy and space utilizations [40]. In particular, reefers are designed to distribute cold air from the floor via specific T-shaped decking (Figure 5). Hence, it is crucial that package design provide vent holes to facilitate vertical airflow inside a reefer [16,17]. 

### 2.5. Refrigerated Display Cabinets

**Figure 6:**
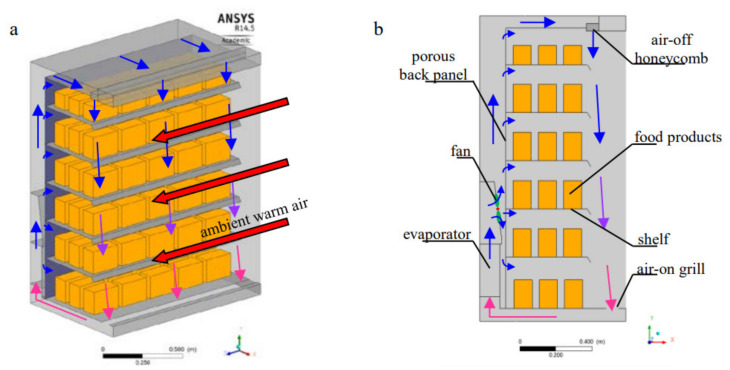
Illustration of the structure of the conventional refrigerated cabinet: (**a**) 3D structure; (**b**) 2D structure [41]. Reprinted with permission.

Fruits and vegetables should be kept under optimum temperatures all the time. Radiation and other sources of heat should be minimized during sales in stores and supermarkets. Retail (display) cooling systems accomplish this by employing a specialized refrigeration technique that allows good visibility and ensures free access to stored food for shop customers using a virtual insulation barrier called an air curtain [41]. An air curtain is developed by circulating the air from the top to the bottom of the case (Figure 6). The air curtain is a non-physical barrier between the cool air inside the case and the warm shop environment. This air passes over all the food products, resulting in heat transfer from the food to the air, which keeps the produce at a predefined temperature. Simultaneously, heat is also transferred from the shop environment to the air curtain. This causes the temperature of the air curtain to increase and reduces the effectiveness of the air curtain in the lower compartments of the display case. To this end, display cabinets are characterized by their large consumption of electrical energy because of their direct interaction with the ambient environment. Hence, the design of an energy-efficient display cabinet has been subject to active modeling and analysis. 

A 3D CFD model was used to assess the effect of air curtain velocity, width, discharge angle, and positioning of the air curtain outlet from the front edge of the top shelf, etc. Moreover, it is important to quantitatively determine the amount of the air curtain reaching the bottom of the case to be cooled and the amount that escaped into the shop environment. This is important to optimize energy usage and reduce the discomfort of the consumer [18]. Hence, the study of air curtains is required because these can be easily disrupted by air circulation in front of the cabinet or by consumers taking food from the shelves [18,19,41].

### 2.6. Objectives of Mathematical Modeling in the Fruit Cold Chain

The objective of mathematical modeling in the produce cold chain is to provide qualitative and quantitative data for a better understanding and interpretation of the produce–environment interaction. This information is used to make predictions about produce shelf life for efficient and cost-effective postharvest loss prevention. Energy-saving and material usage are crucial aspects of the produce cold chain to protect the natural environment for sustainability [15]. The analysis, design, and optimization of these systems, in experimental conditions alone, is time consuming and expensive due to many complex factors. Hence, mathematical models are a valid alternative to expensive and difficult experiments [15,16,17,38,39]. 

Many studies involved the 3D airflow velocity field, the spatiotemporal temperature distributions, cooling rate, cooling uniformities, and refrigeration heat loads of stacked produce [12,13,14,15,16,17]. Such models are used to predict the precooling time, identify hot spots and cold spots in the cooling environment, and calculate energy utilization to optimize the operation of the cooling process. Package design, mechanical stability, and safety are investigated using CSD models. 

## 3. Computational Fluid Dynamics in the Produce Cold Chain

The precooling, cold storage, refrigerated transport, and display units in the postharvest period are, fundamentally, heat and mass transfer processes studied using the science of fluid mechanics. CFD is a science that, with the help of digital computers, produces quantitative predictions of fluid flow phenomena based on conservation laws, presented as a set of coupled differential equations called Navier–Stokes equations. These sets of equations describe how the velocity, pressure, temperature, and density of a moving fluid are related. However, these equations are too difficult to solve analytically, and thus approximations and simplifications have been made to provide the Reynolds-averaged Navier–Stokes equations (or RANS equations). Equations (1)–(3) are the general form of the RANS equations for the conservation of mass, momentum, and energy, respectively. Usually, high-speed computers are used to solve these equations using a variety of numerical techniques such as finite difference, finite volume, and finite element methods.

(1) ∇·U=0

(2) $$\frac{\partial U}{\partial t}+\nabla \cdot \left(U⊗U\right)=\nabla \cdot \left(\left(\mu +\mu_{t}\right)\nabla U\right)+S_{U}-\nabla p$$

(3) $$\rho_{a}C_{pa}\left(\frac{\partial T_{a}}{\partial t}+U\cdot \nabla \cdot T_{a}\right)-\nabla \cdot \left(\left(k_{a}+k_{t}\right)\nabla T_{a}\right)-Q=0$$

where ***U*** is the vector of the velocity (m s^−1^), *t* is time (s), *µ* is the dynamic viscosity of air (kg m^−1^s^−1^), *µ_t_* is the turbulent eddy viscosity (kg m^−1^s^−1^), *p* is pressure (Pa) causing the fluid flow and *S_U_* (m s^−2^) is momentum source inside the fluid domain, *C_pa_* (J kg^−1^K^−1^) is the heat capacity of the air, *⍴_a_* (kg m^−3^) is the density of the air, *T_a_* (K) is the air temperature, *k_a_* (W m^−1^K^−1^) is the thermal conductivity of the air, and *k_t_* (W m^−1^K^−1^) is the turbulent thermal conductivity. The turbulent thermal conductivity is a function of the turbulent eddy viscosity, the heat capacity, and Prandtl number of the air. The turbulent eddy viscosity is calculated by the turbulence model. 

Turbulence modeling is a key subject in CFD simulations. There are several formulations for solving turbulent flow problems. The most widely used are the k-ε and the k-ω eddy viscosity models. Another very popular turbulence model that has proven to be very successful in different applications is the Shear Stress Transport k-ω (SST k–ω) model, which combines the best aspects of the k-ε and k-ω models. Most frequently, the SST k–ω turbulence model is used in modeling flow in cold postharvest handling systems [15,39]. Solving these equations requires numerical methods that involve the study, development, and analysis of algorithms for obtaining numerical solutions. Due to the complexity of the problem, a computer program or code is written to communicate the algorithms to a high-speed computer. There are many choices of computer codes in the science of numerical analysis.

### 3.1. CFD Solution Procedures

#### 3.1.1. Problem Formulation

Predicting the airflow, heat, moisture, and gas distribution in the various cold handling systems (precooling, cold storage, and refrigerated transport) is required. This information is then used to examine the produce quality keeping, energy, material, and space usage of postharvest handling processes. The airflow distribution is generally formulated as a steady-state problem, assuming time-invariant flow conditions, whereas the heat transfer and other gaseous and particulate transport phenomena are transient problems [14,15,39].

#### 3.1.2. Geometry and Flow Domain

**Figure 7:**
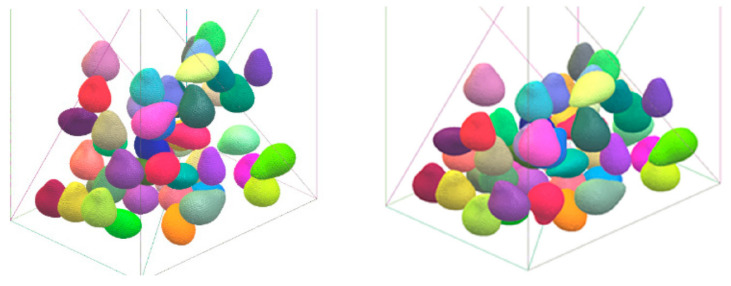
An example of geometries representing produce distribution in a box. Two snapshots showing how the random packing of shapes occurs in the discrete element modeling approach. Progress in the model occurs until the shapes can no longer “settle”, which means that no shape will move further under the influence of gravity alone [44].

Developing the geometry of the produce and the flow domain is a crucial step in CFD modeling. The development of a model that closely accounts for the true shape and details of the produce stack poses a key challenge in the research and design of postharvest processing equipment [15,16,17,39]. Traditionally, as in many other applications, geometries and flow domains were simplified as 1D or 2D geometries for fast and feasible computations. During the last decade, however, computing capacity and solving codes have been improved, and considering more detailed and realistic 3D shapes became possible. Fruit may be dumped into a packaging box randomly, without any pattern. In this case, the fruit are spatially distributed at random inside the box. The geometry of randomly loaded fruit in a box is generated using a discrete element model (DEM) for nearly spherical fruit [15] and for non-spherical fruit such as pear [42,43,44]. The DEM method is a numerical technique for solving Newton’s equations of motion of an assembly of interacting particles (Figure 7). The forces accounted for are gravity and contact forces, due to collision with other spheres or walls. Contact forces are described by a contact force model. This is a useful tool to obtain the three-dimensional array of elements of different sizes that can be used to develop the model geometry. When the fruit is stacked in an orderly manner inside the box, the geometry can be easily developed by manually using the graphic tool of the CFD software package. 

**Figure 8:**
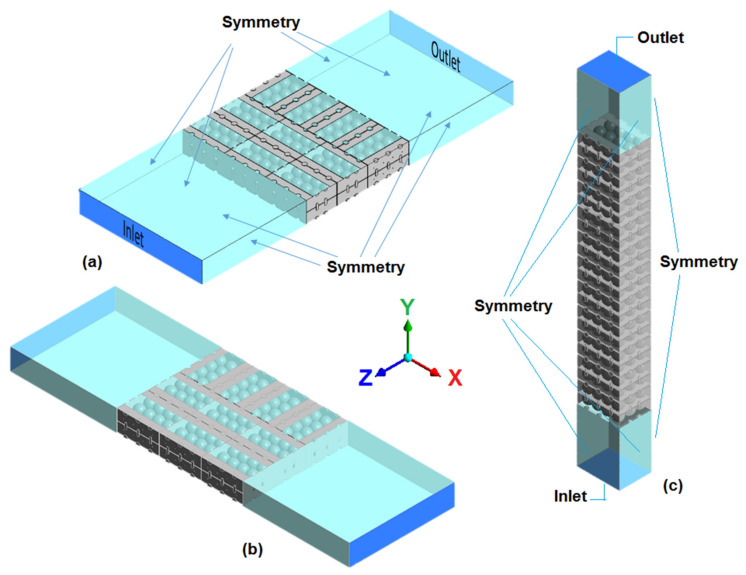
Illustration of the geometry of produce-cooling configurations. (**a**) a layer with its long side perpendicular to the horizontal airflow direction, (**b**) a layer with its short side perpendicular to the horizontal airflow direction, both mimicking precooling and room-cooling operations and (**c**) individual vertical column of stack along the vertical airflow direction, mimicking the cooling airflow in refrigerated containers.

Figure 8 illustrates the geometry of various produce precooling structures. A forced air-cooling (FAC) system is modeled as a flow-through packed bed with the fruit stack placed in a rectangular tunnel. This system is the subject of active research in the CFD analysis of flow through an immobilized packed bed [12,13,14,15,16,17]. The system arises from a methodical simplification of the actual system to reduce computational complexity. Figure 8a,b illustrates airflow through a single horizontal layer taken out of a pallet of produce under forced air precooling or room-cooling conditions. Similarly, Figure 8c uses a single vertical column for vertical airflow configuration (typical in reefer containers). These simplifications have been shown to be sufficiently accurate by several researchers in the area [12,13,14].

**Figure 9:**
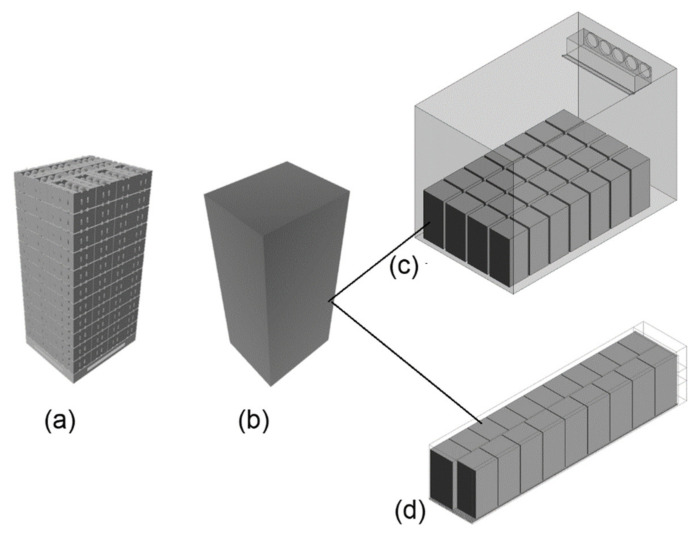
The porous medium modeling approach. Stacked and palletized fruit (**a**), porous medium model of a pallet (**b**), porous replica of a pallet placed in a cool storeroom (**c**), and in a refrigerated transport container (**d**).

Recently, with the advent of high-speed computing machines, it becomes possible to model the entire distribution of fruit inside a pallet. Such a model is invaluable to obtaining a detailed analysis of the effects of box vent designs, box orientation, and arrangement [39]. However, the incorporation of the detailed geometries in the model of a fully loaded cold storage room or reefer container is still impractical. The geometric complexity of large and complex systems such as cold storage rooms and reefer containers are avoided by applying the method of porous media approach. In this approach, a pallet of fruit (Figure 9a) is considered a porous domain (Figure 9b) and incorporated into the cold storeroom (Figure 9c) or reefer container (Figure 9d) models.

#### 3.1.3. Establishing the Boundary and Initial Conditions

The FAC system for precooling of fresh fruit is generally modeled as the flow in a tunnel with the stacked fruit placed between an input boundary (at a static pressure of 0 Pa) and an output boundary with a negative (suction) pressure (see Figure 8a,b). The domain of the fruit stack is separated from the fluid domain, creating the air–solid interface (no-slip wall) boundary where the heat, moisture, and gas exchanges are taking place.

Cold storage room and reefer containers are modeled as closed systems with an internally located air-driving fan. The cooling unit houses the evaporator fan of the refrigeration system. The fan blows the return air through the evaporator coil and creates the overall air circulation in the room. This unit is frequently modeled as a separate domain with a heat sink term, to account for the refrigeration effect of the cooling medium, and a momentum source term, to account for the air-driving effect of the fan of the cooling unit (see Figure 4). In the porous domain, the fluid flow and heat transfer equations are solved by applying the volume-averaged RANS equations [15,16,17]. The mass conservation (continuity) and momentum conservation in the porous domain are given by Equations (4) and (5), respectively, assuming constant porosity in space.

(4) ε∇·U=0

(5) $$\frac{\varepsilon \partial \left(U\right)}{\partial t}+\varepsilon \nabla \cdot \left(\left(U⊗U\right)\right)-\varepsilon \nabla \cdot \left(\left(\mu +\mu_{t}\right)\nabla U\right)=\varepsilon S_{U}-\varepsilon \nabla p$$

The fluid (air) and solid (fruit) phase heat transfer phenomenon is modeled using Equations (6) and (7), respectively,

(6) $$\varepsilon \left(\left.\rho_{a}C_{pa}\right)\right.\left(\frac{\partial T_{a}}{\partial t}+U\cdot \nabla T_{a}\right)=\varepsilon \nabla \cdot \left(k_{aeff}\nabla T_{a}\right)+\varepsilon q_{a}+h_{sa}\left(T_{s}-\left.T_{a}\right)\right.$$

(7) $$\left(1-\varepsilon \right)\left(\left.\rho_{s}C_{ps}\right)\right.\frac{\partial T_{s}}{\partial t}=\left(1-\varepsilon \right)\nabla \cdot \left(k_{s}\nabla T_{s}\right)+\left(1-\varepsilon \right)q_{s}+h_{sa}\left(T_{a}-\left.T_{s}\right)\right.$$

where *C_pa_* (J kg^−1^K^−1^) is the heat capacity of the air, ρ_a_ (kg m^−3^) is the density of the air, ρ_s_ (kg m^−3^) is the density of the apple fruit, *T_a_* and *T_s_* (K) are the air and produce temperatures, respectively, *k_aeff_* (W m^−1^K^−1^ ) is the effective thermal conductivity of the air, *C_ps_* (J kg^−1^K^−1^) is the heat capacity of the produce, *k_s_* (Wm^−1^K^−1^) is the thermal conductivity of the produce, and *q_s_* (W m^−3^) is the produced heat generation.

#### 3.1.4. Mesh Generation

A crucial stage of CFD application is grid generation. In CFD, grid generation undertakes the partitioning of the model geometries (fluid domains and the solid domains) into several nonoverlapping subdomains called computational grids [45,46]. The grids must capture the changes in the geometries of the system to be modeled, as well as all changes in the flow, with sufficient accuracy. Depending on the shape and complexity of the geometry, the flow domain grids can be structured, unstructured, or hybrid. For reasons of computational accuracy and efficiency (convergence rate), one should try to avoid extreme cell side ratios and skewed angles in individual cells [46]. A hybrid grid contains a mixture of structured portions and unstructured portions. This can help in optimizing grids in complex geometries. Parts of the geometry with regular structure can have structured grids, and irregular structures can have unstructured grids. Due to the relatively complex geometry of packed fruits, unstructured or hybrid grids are commonly used in the CFD modeling of cold handling systems for the postharvest period.

#### 3.1.5. Pre-Processing

During pre-processing, the values of the input parameters, including produce property data, boundary conditions, initial conditions, momentum source, and the capacity of refrigeration, are specified. The simulation strategy is also established. The strategy for performing the simulation involves determining such things as the use of space marching or time marching, the choice of turbulence or additional variable models, and the choice of algorithms. Space marching and time marching are assessed in close consideration of the grid sensitivity analysis. The time-marching and the space-marching methods provide comparable results if care is taken in selecting the appropriate mesh size near the body surface. 

#### 3.1.6. Simulation and Post-Processing

The simulation is performed with various possible options for interactive or batch processing and distributed processing. The simulation generally starts from an initial solution and uses an iterative method to reach a final solution. As the simulation proceeds, the solution is monitored to determine if a “converged” solution has been obtained. CFD solvers are progressively taking advantage of the new parallel computing hardware using massive multicore architecture. Clusters with hundreds of thousands of cores are becoming the standard in the modern high-performance computing (HPC) world. For instance, [39,47,48] performed detailed modeling of the airflow and heat transfers within a pallet of packed fresh fruit and characterized the heterogeneities in cooling and quality evolution, using 25.4 × 10^6^ cells. The new features resulted in increased performance without sacrificing accuracy. To this end, recent CFD studies of the postharvest period have demonstrated considerable improvement.

Post-processing involves extracting the desired flow properties and visualizations such as contour plots, vector plots, streamlines, and line plots. Quantitative data extraction from individual nodes, on boundaries, and along lines and points can be implemented. Switching between time step results can be made to create animations. The computed flow properties are then compared to results from experimental studies to establish the validity of the computed results. 

### 3.2. Overview of CFD Software

CFD simulation uses 3D CAD tools for geometry creation, a mesh generator, solver software to run the simulation, and visualization software for post-processing. Generally, commercial software packages that incorporate all these software and functionalities are used in postharvest applications [15,16,17]. Open-source CFD solvers are less frequently used in postharvest applications. However, these alternatives, in addition to being free to use and distribute, provide users the license to modify the source code as needed. The most used general-purpose codes in the postharvest period are summarized in Table 1.

## 4. Notable CFD Studies 

### 4.1. Airflow and Temperature Distribution during Precooling of Produce

Table 2 summarizes studies on airflow and temperature distribution of the fresh fruit precooling process. Several crops have been studied using the CFD approach, including apples, pomegranate, citrus, kiwi, strawberry, tomato, etc. However, apple fruit is the most-studied fruit. There are also studies conducted on artificial spherical balls mimicking fruit [59,60]. Such generalized spherical artificial fruits are only used to investigate airflow distribution. Model geometry considered in the CFD model of the produce precooling ranges from individual fruit [51] to an entire pallet stack of fruit [13,14,43,47]. In between, we have studies on individual packages [61,62], the horizontal layer out of a pallet [13,63], or the vertical column out of a pallet [39,48].

In a typical analysis, [59,60] studied packaging boxes are designed with a range of vent hole sizes, shapes, and positions, with respect to cooling rate, cooling uniformity, and pressure drop. Clearly, the higher the vent area, the better the cooling rate and cooling uniformity. However, beyond a certain limit, increasing the vent hole proportion has no significant benefits on the cooling rate and causes problems in stack statics. This limiting value was reported to be 7% [60]. In another study, the cooling characteristics of existing package designs and package accessories such as liners were analyzed and characterized through a detailed quantification and visualization of the airflow, pressure drop, and temperature distributions across the stack [13]. Here, the authors demonstrated that liners had a strong effect on cooling rate and delayed cooling time by factors of three compared to stacks with no liners.

The cooling of strawberries is very crucial since the shelf life of this product is relatively short; the fruit is a high-value crop and is one of the most perishable fruits. Strawberries are susceptible to mechanical damage, microbial decay, and water loss. If strawberries are left without cooling for a few hours, their quality will be reduced to an extent that a proportion of marketable fruit is lost. Hence, strawberries need stricter temperature management than many other fruits. Through properly executed temperature control, the microbial activity and the respiration process can be reduced. Wilting and shriveling due to moisture loss can also be reduced. Ferrua and Singh [61] undertook CFD analysis of the FAC process of retail packages of strawberries. The authors clearly demonstrate that the design of the packaging system significantly influenced the uniformity of the cooling process. Here, the airflow path through clamshells was visualized. The evaluation of the flow path obtained from the CFD simulation helped the researchers easily identify bypass flows. Nalbandi et al. [64] used experimental and modeling approaches to design a new package and cooling system for the precooling of strawberries. The CFD model of the airflow through the traditional system helped the researchers perform an in-depth visualization of the airflow path and distribution. The innovative design resulted in faster and more uniform cooling, compared with the traditional system.

Berry et al. [65] studied the effect of carton designs on airflow resistance, cooling rate, cooling uniformity, and energy usage in the precooling of apple fruit. Four carton designs with different vent hole areas were compared. The authors used an experimentally validated CFD model to investigate the airflow resistance, cooling rate, cooling uniformity, and package-related energy consumption of the four designs. Experiments were used to quantify box compression strength and study the effect of vent hole proportion on the mechanical strength of the carton. The study showed an energy use reduction of 58% with a slight change in the vent hole design compared to the standard vent design. 

Defraeye et al. [14] used a CFD model to investigate the cooling performance of an existing package design and two new ones (Supervent and Ecopack). The CFD model helped the researchers to obtain the detailed and closer quantification and visualization of the airflow path. This enables the researchers to identify airflow short circuits of alternative designs. Through the redesign of the vent holes, airflow short circuits were removed, and the rate and uniformity of the cooling process were enhanced. The energy usage, fruit quality preservation, and throughput of the process were also significantly improved by the new design. In a subsequent study, Defraeye et al. [66] further investigated alternatives to forced-air precooling. In this work, the authors used an experimentally validated CFD model to investigate the warm loading of citrus fruit into refrigerated containers for cooling during marine transport for logistic and economic savings. The authors underlined the importance of box design and proper stacking to reduce airflow short-circuits between pallets.

**Table 2:** Objectives, numerical techniques, and results of computational-fluid-dynamics-based analysis in precooling studies.

Fruit	Model Geometry	Numerical Technique	Objectives	Result	Reference
Pomegranate	Pallet	FVM in CFX	Redesigning the vent holes of packaging.	The new vent hole design enabled 14.4% faster cooling and lowered the airflow resistance by 6.5%.	[13]
Apple	Pallet	FVM on Open FOAM	Assessing thermal heterogeneity and the associated differences in quality.	Without precooling, about 23% more quality loss was found than with precooling.	[14,43,47,48]
Pomegranate	Pallet and single layer	FVM in CFX	Analyzing the effect of package design on the rate and uniformity of cooling.	A cooling rate difference of 30% was observed between two commercial package designs. Plastic lining increased the average 7/8th cooling time by threefold.	[13]
Apple	Single fruit,	FVM in Fluent	Studying the effect of air inflow velocities on the apple temperature distribution during forced convection cooling.	Cooling rate increases significantly with air inflow velocity up to 2.5 m/s; any further increase resulted in a relatively low increase in cooling rate.	[55]
Apple	Single package	FVM in Fluent	To evaluate the cooling performances, energy usages, and fruit quality (chilling injury and mass loss) of an existing container and a newly developed container.	A new package with improved cooling performance was proposed. For all existing package designs, the optimal air inflow velocities lay in the range 0.4–1 m/s (or 3–5 L s^−1^kg^−1^).	[62]
Apple	Pallet	FVM in Fluent	Evaluate cooling rate and uniformity, energy efficiency, and fruit quality (including chilling injury and mass loss).	For all existing package designs, the optimal air-inflow velocities lay in the range 0.4–1 m/s (or 3–5 L s^−1^ kg^−1^).	[63]
Kiwifruit	Single layer out of a pallet	FVM in Fluent	To determine the optimum pressure drop and cooling airflow rate for improved cooling rate, energy usage, and process throughput.	A pressure drop of 100 Pa with a corresponding cooling airflow rate of 0.25 L kg^−1^ s^−1^ was proposed as the optimal operating point.	[56]
Citrus	Single layer out of a pallet	FVM in Fluent	To analyze the cooling rate and energy usage of existing container designs and new container designs.	The different containers were ranked with respect to their performances in cooling rate, cooling uniformity. and energy consumption.	[14]
Strawberries	Single layer out of a pallet	FEM in COMSOL Multiphysics	To assess the performance of a new package and cooling system for the precooling of strawberries.	Improvements were made in the uniformity of cooling of strawberry and reduced the fruit decay.	[61]
Apple	Single package	FVM in Fluent	To study the effect of vent shape, vent distribution, and stacking pattern on the cooling quality.	Vertical oblong-shaped vent and triangular vent distribution could improve the longitudinal and lateral airflow.	[67]
Artificial, Spherical Plastic Ball Citrus	Single package to half a pallet	FVM in Fluent	To study the effect of package vent design on airflow and heat transfer.	A 7% vent area proportion is optimum.	[59,60]

### 4.2. CFD Models Analysing Cold Storage Room

Airflow distribution, particulate transport, and gaseous substance distribution inside cold storage rooms are the major problem categories that have been investigated using a CFD model (Table 3). Additionally, the effect of packaging design on the airflow and heat transfer, and the accompanying energy usages of cold storage rooms, have been subjects of interest. Cold storage rooms are modeled as closed systems inside which the cooling air recirculates, extracting heat from the stacked produce and transferring it to the internally placed evaporator unit of the refrigeration system. The finite volume method (FVM) is frequently implemented in commercial software packages such as ANSYS Fluent and ANSYS CFX to model cold storage rooms (Table 3). Due to the relatively complex geometry of the actual system, stacked fruit in cold storage rooms are normally simplified as porous domains, initially at a uniform temperature (7/8th cooling temperature) as it is received from a precooling unit. Usually, the cold storage room is kept under a constant air circulation rate (≈100 h^−1^) and a set point temperature appropriate for the optimum handling of the commodity. The walls, ceiling, and floor of the room are considered a wall boundary with a heat transfer coefficient ranging from 0.4 to 0.7 W m^−2^ K^−1^ [15].

Ambaw et al. [15] used a porous medium CFD model of airflow and temperature dynamics inside a fully loaded cool storeroom of apple fruit. Using this model, the authors analyzed several load-shifting scenarios by cycling the temperature set point between 1.2 °C and 0.6 °C following a day/night regime to reduce energy cost. The air circulation fan was shown to be the major source of heat load. Discontinuous use of the cooling operation, including 12 h on/12 h off, 10 h on/14 h off, and 8 h on/16 h off, was investigated. Using the model, it was possible to assess the produce temperature history and identify the hot and cold zones inside the cool store.

**Figure 10:**
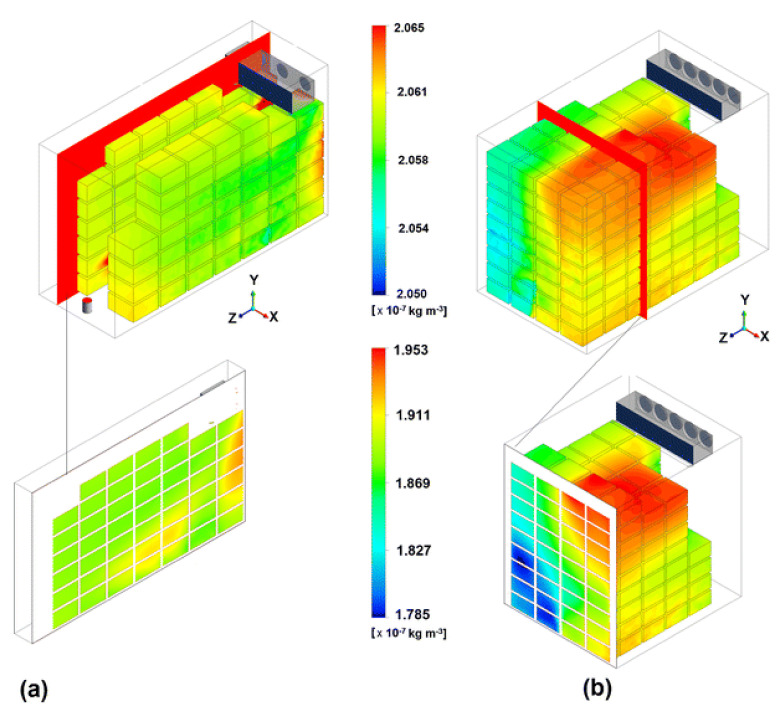
Simulated contour of unbound (top row) and bound (bottom row) 1-MCP at 2 h in CS1 (**a**) and CS2 (**b**). Simulations correspond to treatment at 1 °C and 1-MCP dose of 0.625 ppm [68]. Reprinted with permission.

In another approach, a porous medium CFD model was used to numerically analyze the distribution of 1-Methylcyclopropene (1-MCP) in cool stores for apple fruit [68]. This study performed a detailed analysis of the effects of air circulation, room shape, and bin material on the convection–diffusion–adsorption of the gas in fruit and other non-target solid materials in the cold room (Figure 10). The authors showed that wooden bins deplete 25% more of the active substance than rooms filled with fruit in plastic bins. Delele et al. [69] applied the CFD technique to investigate the effectiveness of postharvest storage fungicide fogging systems. Using this approach, the author investigated the effect of airflow rate and different bin handling parameters on fungicide particle flow, and depositions on fruit surfaces were quantified.

**Table 3:** Objectives, numerical techniques, and results of computational-fluid-dynamics-based analysis in cold storage room studies.

Fruit	Numerical Technique	Objectives	Result	Reference
Apple	FVM ANSYS in Fluent	To investigate the effectiveness of postharvest storage fungicide fogging systems.	The effect of airflow rates and different bin handling parameters on fungicide particles’ flow and deposition were obtained.	[69]
NA	FVM ANSYS in CFX	To analyze the aerodynamic sealing of doorways of refrigerated rooms.	The sealing efficiency was estimated for different situations.	[70]
Citrus	FVM ANSYS in Fluent	To investigate the cooling performance of a partially loaded cold store in the cooling process.	Effect of loading pattern on the cooling process was established.	[71]
Dates	FVM ANSYS in Fluent	To define the suitable precooling conditions leading to homogeneous storage temperature inside the room.	A new cold storeroom design was proposed using specific aerodynamic air deflector profiles.	[72]
Table Grapes	FVM ANSYS in Fluent	To analyze the effects of the packaging components (bunch carry bag and plastic liners) and box stacking on airflow, heat, and mass transfer.	The presence of the carry bag increased the half and 7/8th cooling time by 61.09% and 97.34%, respectively.	[59,60]
NA	FVM ANSYS in Fluent	To evaluate the air infiltration rate through sliding doors.	Air temperature difference between spaces affected the air infiltration. For this case study, the infiltration rate increased by 0.012 m^3^ s ^−1^ per K of air temperature difference.	[73]
Apple Fruit	FVM ANSYS in CFX	To analyze the 1-MCP distribution in commercial cool storerooms: porous medium model application.	New index to visualize heterogeneity in time was presented.	[68]
Apple Fruit	FVM ANSYS in CFX	To analyze energy- and cost-saving alternatives.	The study showed that the air circulation fan is the major source of heat load. Hence, an attempt to reduce energy costs should first consider reducing the fan operation time.	[15]

### 4.3. Refrigerated Container (Reefer)

Table 4 summarizes the general objective, analysis, and results obtained by using a computational modeling approach in the area of refrigerated containers. As in the case for cold storage rooms, the model geometry of a commercial-scale refrigerated container is complex due to the size range of the geometric parts. Hence, the porous medium approach is used for the simplification of the system. Inside the cold store, the cooling airflow direction is mainly horizontal, while in reefer containers, airflow is mainly vertical (from bottom to the top of the stacked produce). To this end, the design of package vent holes and package arrangement should take this into account so that enough vertical airflow is attained. The cooling unit of the reefer container is limited in capacity, and it can only lower the pulp temperature very slowly. Hence, the produce must be precooled to a specified optimum transport temperature prior to loading into containers [14,48]. The understanding of the effects of factors such as ambient temperature, sunlight, heat from the motors of the evaporator fans, packaging, packaging arrangement, produce physiology, heat from defrosting the evaporator coil, etc., is crucial for successful reefer temperature control. The CFD modeling technique has been successfully used to investigate a new cold chain protocol for citrus fruit [39,66]. Using this technique, the authors demonstrated the advantages of the ambient loading of fruit in reefer containers for cooling during long-haul marine transport. The effect of environmental factors such as solar radiation on the energy usage of reefer containers has been studied by incorporating the azimuth angle into the CFD model [74]. 

### 4.4. Moisture Distribution in Cold Storage Room

Modeling the moisture transport phenomena requires modification of the heat transfer equations of the CFD model so that it incorporates the respiration and transpiration processes of the produce. Moreover, the model should incorporate the heat loss/generation due to the evaporation/condensation of water at the surface of the produce. These phenomena are captured by Equations (8) and (9)

(8) $$\left(\rho_{a}\left.C_{pa}\right)\right.\left(\frac{\partial T_{a}}{\partial t}+U\cdot \nabla T_{a}\right)\;=\;\nabla \cdot \left(\left(k_{a}+k_{t}\right)\nabla T_{a}\right)+h_{pa}\left(T_{p}-T_{a}\right)$$

(9) $$\left(\rho_{a}\left.C_{pa}\right)\right.\left(\frac{\partial T_{p}}{\partial t}\right)\;=\;\nabla \cdot \left(k_{p}\nabla T_{p}\right)+h_{pa}\left(T_{a}-T_{p}\right)+Q_{r}-Q_{v}$$

where *h_pa_* is the heat transfer coefficient of the interface between the produce and the cool store atmosphere, *Q_r_* is the respiration heat generation, and *Q_v_* is the heat loss due to the evaporation of water from the surface of the produce. The moisture distribution in air is modeled, as given by Equation (10), to be coupled to the basic fluid flow equations (Navier–Stokes equation)

(10) $$\rho_{a}\frac{\partial G}{\partial t}+\nabla \cdot \left(GU\right)-\left(D_{a}-D_{t}\right)\nabla G)=m$$

where *G* is the moisture concentration (absolute humidity), *D_a_* is the diffusivity of moisture in the air, *D_t_* is the turbulent diffusion coefficient, and *m* is the rate of evaporation of water from the surface of produce into the cool store atmosphere, which is governed by the equilibrium between the room moisture content and the water activity of the produce. The turbulent diffusion coefficient, through the empirical turbulent Schmidt number, is a function of the turbulent viscosity (*D_t_ = μ_t_/Sc_t_*).

For the product phase, the heat generation of the produce and the heat loss/generation, due to evaporation/condensation of water at the surface of the produce, are incorporated into the energy conservation equations (Equations (11) and (12)) as follows:

(11) $$\rho_{p}\frac{\partial }{\partial t}\left(r_{p}\left.H_{p}\right)\right.\;=\;\frac{\partial }{\partial x_{i}}\left(r_{p}\lambda_{p}\frac{\partial T_{p}}{\partial x_{j}}\right)+h_{T}A_{spec}\left(T_{a}-\left.T_{p}\right)\right.+r_{p}q-h_{fg}m$$

(12) $$m=-\frac{\partial M_{p}}{\partial t}\;=\;h_{m}A_{spec}\left(\rho_{p}^{v}\right.-\left.\rho_{a}^{v}\right)$$

### 4.5. Modeling Produce Quality

During the storage of fruit, quality attributes such as taste, firmness, color, and flavor are measured to follow the evolution of quality degradation. The coupling of fruit quality models with the CFD model equations is interesting because such a model can be used for a virtual investigation of the effect of storage conditions on the quality of the produce. This will further make it possible to identify critical operational requirements during postharvest storage and to improve decision-making in the cold chain logistic management and inventory control. However, there are few published studies that incorporate produce quality in a CFD model. Wu and Defraeye [39] incorporated a generic quality model (Equation (13)) into the basic CFD model of airflow and heat transfer inside ventilated cartons for different cold chain scenarios and modeled the quality evolution of individual fruit in a pallet.

Enzyme kinetics is used to quantify produce quality in time. The zero-order kinetic (Equation (13)) is frequently used to estimate produce quality while a commodity passes through the precooling, cold storage, and refrigerated transport stages

(13) $$A_{T}=A_{0T}-k_{T}t$$

where *A_T_* is the fruit quality after storage duration of *t* at temperature *T*, *A_0T_* is the initial quality of the produce at the initial time, and *k_T_* is the rate constant at temperature *T*. The temperature dependence of the rate constant is obtained from the Arrhenius relationship (Equation (14)) [81,82,83] and the *Q_10_* value (Equation (15)), which measures the temperature sensitivity (a measure of the rate of change) of an enzymatic reaction rate or a physiological process due to a temperature increase of 10 °C. The *Q*_10_ coefficient is commonly used in postharvest studies regarding fruit respiratory activity. Enzymatic and physiological processes are two to three times faster for each 10 °C of temperature increase. The range of *Q*_10_ can go from 1 to 10 or more

(14) $$k_{T}=k_{0}e^{-\frac{E_{a}}{RT}}$$

(15) $$Q_{10}=\frac{k_{T+10}}{k_{T}}=e^{\frac{E_{a}}{R}\left(\frac{1}{T}-\frac{1}{T+10}\right)}$$

where *k*_0_ is the so-called “pre-exponential factor” [d^−1^], *E_a_* the activation energy [J mol^−1^], *R* the gas constant [8.314 J mol^−1^ K^−1^], and *k_T+10_* and *k_T_* are the rate constants [d^−1^] at temperatures *T* and *(T +* 10) [K], respectively.

## 5. Mechanical Strength of Corrugated Fiberboard Cartons in the Produce Cold Chain

### 5.1. Compression Strength

The compression strength of CFCs is affected by many factors, including box dimensions, corrugated box flute configuration and sizes, the basis weight of linerboards/medium, temperature, humidity, stacking configuration, transportation, and handling conditions. Stack misalignment significantly contributes towards a reduction in carton compression strength [84,85]. Refrigerated conditions (0 °C and 90% RH), when compared to shelf-life conditions (23 °C and 50% RH), reduced the compression strength of corrugated cartons—about 16% [86]. At refrigerated conditions, the water content of paper material increases significantly, breaking the bonds between cellulose fibers by increasing the moisture content [87]. The effects of different storage conditions (−0.5 °C at 90% RH; 4 °C at 90% RH; 10 °C at 90% RH) on the mechanical performance of two types of ventilated cartons (“Supervent” (4.7% vent area) and “Standard’ (3.1% vent area)) were investigated by Pathare et al. [88]. Irrespective of the carton type, reduction in compression strength was maximum at a storage temperature of 4 °C. The authors observed a lower compression strength for the Supervent carton, presumably attributed to the higher vent area on the carton. Fadiji et al. [86] and Singh et al. [89] also reported a linear relationship between the total vent area and reduction in carton compression strength.

Fadiji et al. [24] investigated the creep behavior of ventilated cartons for a 12-hour duration and revealed that load, carton design, and environmental conditions had a significant influence on carton creep rate. According to this study, higher loads resulted in an increased creep rate. Moreover, cartons with vent holes resulted in a higher creep rate than ventless cartons. In comparison with experimental creep strain, the Norton–Bailey creep law and the Power law models showed good correlations. There are different studies on the creep of CFC components such as paper and paperboard [90]; however, the creep of cartons has not been extensively assessed. Further research is required to establish a widely accepted testing protocol for CFCs to understand the time-dependent properties of cartons in real-life situations to enhance and optimize carton design.

Carton liner thickness had a linear relationship with the carton compression strength [27]. The increase in material thickness elevated the buckling of the paper face sheet, thereby reducing the strength of the carton [91,92]. Csavajda et al. [93] evaluated the effect of creasing lines on the compression strength of cartons, identifying the carton strength changes at different creasing points. Cartons with no creasing lines and the smallest height had the least significant reduction in compression strength. Further, carton size, the number of creasing lines, and their interaction significantly influenced the compression strength of the carton.

More recently, the capability of digital imaging to study the deformation of CFCs during compression loading has been revealed. Kueh et al. [94] used 3D digital image correlation (DIC) to examine the contributions to the displacement of panels of compressed cartons. A majority of incidences of vertical displacement of cartons are due to the top and bottom flap crease folds compressing. Moreover, compression of the flaps and horizontal creases dominates the shortening of the carton throughout the compression test. Fadiji et al. [95] evaluated the displacement field of ventilated cartons under compression loading using 3D DIC. Initiation and development of the buckling behavior of the carton panels during compression was shown, and the displacement observed was largely heterogenous. Applying this technique in the carton compression test offers prospects for improved CFC design.

Additionally, the interactions between carton and fruit under compression loading have been reported [96,97,98]. The susceptibility of apple fruit to compression damage in ventilated cartons was studied by Opara and Fadiji [92]. Compression force application on the carton caused apple bruise damage, thus reducing the fruit quality. According to Rodriguez et al. [99], compression damage can trigger ethylene production in fruit, which may affect the respiration rate and subsequent fruit softening, peel injury, and chemical changes [100]. Furthermore, compression damage influences the levels of sugars, organic acids, volatiles, and phenols in fruit [100,101,102,103]. Table 5 summarizes some recent studies on the compression strength of CFCs.

### 5.2. Impact Strength

Carton impact strength is the amount of energy it can withstand under a load or when dropped. Impact, also known as shock or drop, may occur in a range of distribution environments such as the manual or poor handling of cartons, dropping of cartons, falling from a forklift, sudden brake, and the acceleration of transport systems [22]. In the postharvest handling of fruits, it is usual to apply some level of protection against shock to prevent fruit damage. Impact/drop tests are used to measure the ability of the carton to retain and protect the packed produce from freefall. Here, the potential energy of the carton is determined by the product of the weight of the packed produce and the drop height [113]. The vertical distance between the release point of a carton under the influence of gravity onto an impact surface is known as the drop height. Equivalent drop height (EDH) is used to describe the imposed loads on cartons during handling and is defined as the height of freefall needed to produce a similar total velocity change as measured on the shock waveform [20,114]. Equation (16) shows the correlation between EDH and velocity change for an ideal freefalling carton

(16) $$\Delta V=\left(1+e\right)\sqrt{2gh_{EQ}}$$

where g is the acceleration due to gravity, e is the coefficient of restitution, ΔV is the change in total velocity, and $h_{EQ}$ is the EDH.

The percentage of bruised apple fruit in single-walled and double-walled corrugated cartons increased with an increase in drop height, with less damage observed in the upper layer than in the lower layer [115]. Additionally, the pressured area of apples in the single-walled cartons was greater than that in the double-walled cartons. Similarly, Fadiji et al. [116] assessed the susceptibility of apple fruit packed inside ventilated cartons to impact damage. The induced force on the carton resulted in fruit bruise damage and consequent reduction in fruit quality. The carton design and drop height significantly affected the incidence and susceptibility of apple bruise damage. The fruit bruise damage increased with an increase in drop height. Apple fruits at the bottom of the carton experienced greater damage compared to fruit place at the top. The authors suggested the placement of force-absorbing material at the bottom of the carton as an economical way to reduce damage incurred by the fruit. Fernando et al. [117] showed that impact load caused severe neck injuries in packed bananas, which became worse with increasing height. Contrary to the reports by Lu et al. [115] and Fadiji et al. [116], the highest neck injuries, approximately 94% and 91% at heights 30 cm and 50 cm, respectively, were concentrated on bananas in the top two layers inside the carton. The manual handling of cartons was established as one of the major reasons for neck injuries in bananas. Hence, it is necessary to handle cartons and packed produce with adequate care, particularly during palletizing and stacking to reduce the tendencies of damage.

### 5.3. Vibration Strength

Vibration is a prominent cause of mechanical damage, particularly to fruit packed in CFCs [118,119]. Vibration mostly occurs in the distribution chain during transportation and handling. Both the carton and packed produce should be able to endure vibration hazards during these operations [120]. Several factors, such as traveling speed, number and load of axles, truck suspension, and road roughness, affect CFCs exposed to vibration [20,121,122]. Simulating vibration in a laboratory environment can be grouped into three categories: repetitive shock (fixed displacement), random vibration, and multi-axis vibration [120]. Repetitive shock, although widely conducted, is not considered a viable method to simulate actual transport vibration. It is performed on a mechanical shaker table where a system of cams moves the platform in a circular motion [123]. The most common method for simulating transport vibration is random vibration. Here, the power spectral density (PSD) profile is usually of interest, and fast Fourier transformation (FFT) is used to produce these profiles. The profiles represent the energy or power of the vibration excitations. They help to determine the vibration energy in a transit passage and serve as an essential tool in assisting the simulated vibration condition in a laboratory [118]. Vibration damage on packed produce is a consequence of the energy absorbed during transportation. For most packaging applications, power density is obtained using Equation (17) [118,124]

(17) $$PD=\frac{1}{BW}\sum \frac{RMS\;gi^{2}}{N}$$

where RMS gi is the root mean square acceleration measured in g at any instance within a bandwidth (BW) of frequencies and N is the number of samples for a given segment of vibration history.

Transport vibration is not limited to vertical motion alone; therefore, the multi-axis vibration method is useful in obtaining information in other axes of interest. This method provides information and a better understanding of the influence of lateral, longitudinal, pitch, roll, and yaw movements on the packaging response [120,125]. Usually, simulating vibration damage is performed following standards such as the American Society of Testing and Materials (ASTM) or the International Safe Transit Association (ISTA) [126,127]. 

**Figure 11:**
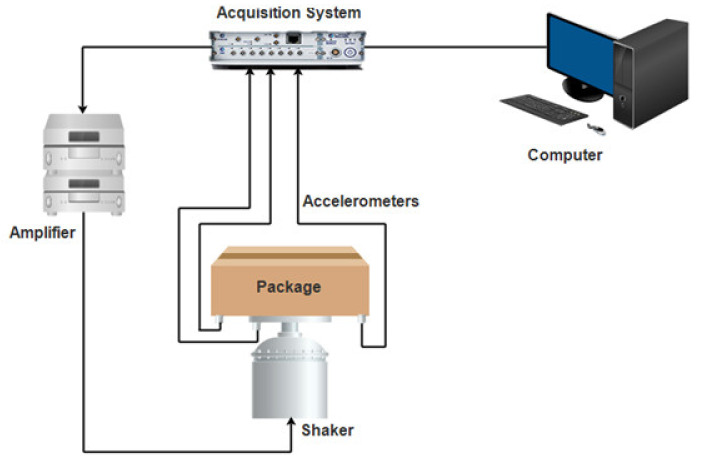
Schematic diagram showing a packaging simulation setup [131]. Reprinted with permission.

Several variables such as frequencies, acceleration, and duration have been used singly or combined as input parameters to drive the simulator for vibrating packages and packed produce [20,118,128,129,130]. Figure 11 shows a schematic illustration of a laboratory setup for package simulation. The major constituents of the simulator are the controller and shaker. Here, the input parameters (for example, frequency, amplitude, etc.) are adjusted accordingly, depending on the intended effect on the packaging and/or contents.

Vibration damage to fresh fruit packed in CFCs often occurs at the vibration resonance frequency; hence, performing assessment across a range of frequencies is very important. In a stacked CFC, resonance was shown to occur in a range of frequencies between 8–18 Hz, which is also found in a transport environment [118,130]. This results in critical dynamic stress and damage to the produce [132]. Table 6 shows some recent examples of the simulated vibration of packed produce. Vibration damage in fruit occurs when the produce experiences acceleration greater than gravity acceleration. The packed fruit does not move with respect to the carton or nearest fruit when the acceleration is below this level.

Park et al. [92] showed that for CFCs under vibration, properties such as vibration transmissibility, resonant frequency, damping ratio, and maximum dynamic stress are relevant for determining the protection attribute of the packaging during transportation. The potency of two CFC designs to protect apple fruit was evaluated by Fadiji et al. [131] using simulated vibration. Carton design and frequency influenced the packaging transmissibility, incidence, and severity of apple damage. Fruit at the top of the carton was more susceptible to vibration damage than fruit placed at the bottom. Similarly, Fernando et al. [119] evaluated the protective performance of different packaging: one-piece and two-piece corrugated paperboard cartons and reusable plastic crates (RPC) for bananas, using simulated vibration. The authors revealed that vibration transmissibility, damping properties of the packaging, the freedom of movement of the fruits, and the packaging construction material influenced the extent of the damage. The one-piece cartons performed best in reducing the banana damage levels caused by in-transit vibration. Produce losses due to vibration damage can be minimized through restricted produce movement, cautious handling, and proper packaging [119,142,143].

### 5.4. Modeling the Mechanical Strength of CFCs

Finite element modeling (FEM) has emerged as an alternative to experimental methods for the mechanical analysis of CFCs [144,145,146]. The elementary matrices are assembled into a global matrix equation that represents the entire structure, as shown in Equation (18) [30,147]

(18) $$\left[K\right]\left\{D\right\}=\left\{F\right\}$$

where $\left\{F\right\}$ is the external force vector, $\left\{D\right\}$ is the displacement vector, and $\left[K\right]$ is the global stiffness matrix. In the post-processing step, raw data generated from the analysis can be viewed graphically. 

**Figure 12:**
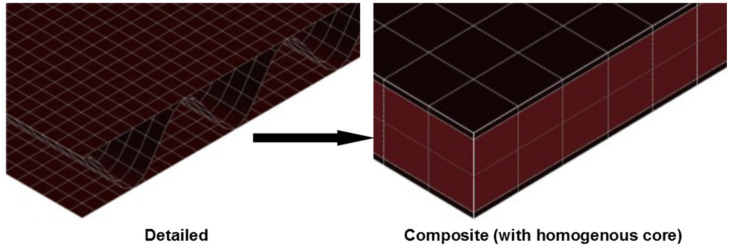
Illustrating the transition of corrugated core to an equivalent homogenized core [27]. Reprinted with permission.

Its popularity as a design tool to evaluate the performance of existing and new designs of CFCs has increased over the years. This is because, when validated, it allows for evaluation without reliance on prototyping and robust experimental analyses. Due to the structural complexity of CFCs, using a detailed geometry increases the difficulty of the analysis and consumes a lot of computational time. For instance, a very fine mesh would be required to model an entire structure with the detailed geometry of the corrugated core, ultimately resulting in a very computationally expensive model [148,149]. To this effect, modeling CFCs by developing equivalent material models of corrugated fiberboard using a homogenization approach has been employed by different researchers [27,86,98,150,151,152,153,154]. This involves a transition of the corrugated core in a fiberboard to an equivalent homogenized core, as shown in Figure 12 [27]. Here, a constant shear strain and stress is assumed through the thickness of the core, and, hence, it is important to reduce the transverse shear moduli by a shear correction factor. This is required to account for the excessive amount of shear strain energy produced.

**Figure 13:**
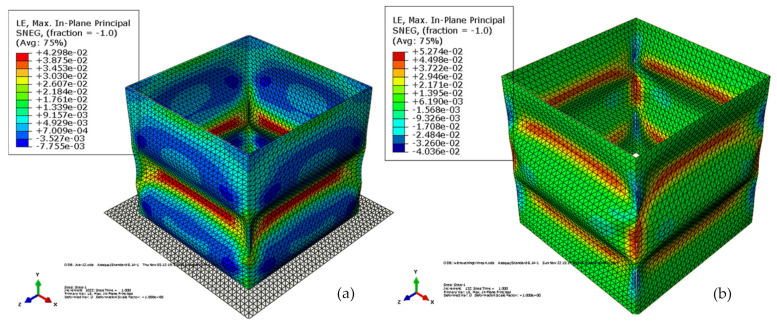
Total strain of the (**a**) creased and (**b**) uncreased cartons [155]. Reprinted with permission.

**Figure 14:**
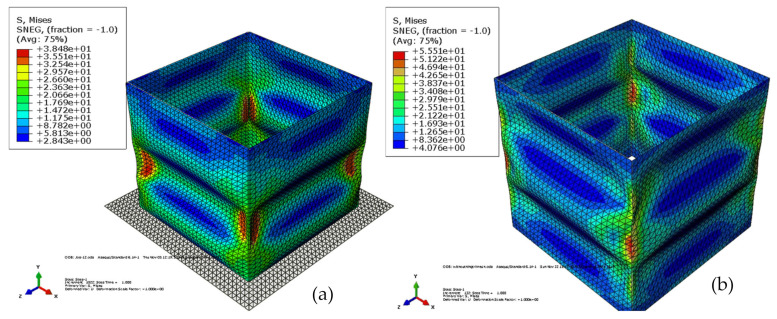
Von Mises stresses of the (**a**) creased and (**b**) uncreased cartons [155]. Reprinted with permission.

FEM can successfully predict different mechanical behaviors of CFCs. These include the compression, buckling, collapse, stability, impact, drop, and including the effects of complex designs on the performance of the carton, etc. Some recent examples of the application of FEA to predict the compression of CFCs are shown in Table 5. Fadiji et al. [86], using FEM, studied the compressive performance of ventilated CFCs by modeling the corrugated paperboard as orthotropic, three-ply laminate with a homogenous core. The authors determined the probable buckling shape and the critical buckling load of the cartons using linear buckling analysis. The effects of vent geometry on the carton compression strength were assessed. Carton compression strength was affected by vent number, size, orientation, and shape. Experimental analyses were used to validate the model results and good agreement was reported. The structural behavior of ventilated CFCs subjected to compression load was studied by Fadiji et al. [27] using FEM by considering the geometrical nonlinearities of the carton. From the contact FEA model, maximum stress concentration was observed at the corners of the carton. Paperboard thickness showed a significant effect on the carton strength. The study emphasized that the constitutional relationship between carton materials and detailed geometrical nonlinearities would enhance the development of models with different configurations for improved carton designs. Consequently, Fadiji et al. [148] used validated FEM models to evaluate the performance of ventilated CFCs by considering the influence of different geometrical configurations of vent and paperboard grade. The authors noted a significant interaction between paperboard grade, carton design, and vent hole design. Zaheer et al. [155] modeled paperboard as an orthotropic elastoplastic material to analyze the influence of compressive load on CFCs using FEA. The model defined the plastic behavior of the carton using Hill’s yield criterion and isotropic hardening. To obtain the critical load and buckling of the cartons, Eigenvalue analysis was performed. Cartons with creases were shown to resist total strains and stresses, compared to cartons without creases as shown in Figure 13 and Figure 14, respectively. Creases on a carton were found to enhance its load-bearing capacity.

Hammou et al. [153] used an efficient homogenization model implemented into the FEM ABAQUS software to study the drop impact of CFCs containing different foam cushions. The model represented the corrugated paperboard with a 2D plate. The homogenization permitted the global rigidities for the equivalent homogenous plate. More damping effect to the shock response of the packed product was observed on cartons with corner foam cushions. Model results were in good agreement with experimental data. Luong et al. [156] proposed a finite element model to study the behavior of CFCs subjected to shocks. The damage boundary curve (DBC) was defined for the cartons, and an elastoplastic homogenization model was developed for the corrugated paperboard. The model predicted the influence of impact dynamics on the structural stability of the cartons, especially in the early stage of design development. A strong correlation between the packed product drop height and the change in velocity the product will experience during handling and distribution was highlighted. Recently, using an elastoplastic homogenization model implemented in ABAQUS FEM software, Luong et al. [157] evaluated the repetitive shock-induced damages on CFCs. It was observed experimentally and numerically that for low-cycle fatigue, carton damage occurred at the first shock, while for limited endurance fatigue, the carton damaged after several shocks.

Despite the successful application of FEM in the mechanical analyses of CFCs, a challenge is encountered in obtaining the mechanical properties of the carton materials used as input parameters in the model. Some of these parameters include Young’s modulus, shear modulus, Poisson’s ratio, and thickness. These are typically obtained from experiments or could be estimated [145,158]. However, due to the hygroscopic nature of paper material, it is constantly changing with factors such as temperature and humidity to reach equilibrium with its environment [22,159]. Additionally, modeling and experimental analyses of CFCs have been solely focused on single cartons. It will be worthwhile to direct further research towards extending these analyses for stacked cartons with or without produce, mimicking the cold chain environmental conditions.

## 6. Model Validation

CFD models must be validated before being used to perform analysis studies to compare scenarios or in any decision-making design steps. The main objective of CFD model validation in postharvest applications is to quantify confidence in the accuracy of airflow and temperature predictions under certain assumptions so that it is used with acceptable levels of uncertainty and error. The level of accuracy required from a CFD analysis depends on the desired use of the results. For qualitative information, such as the profile of the flow field for understanding the behavior of the flow field on a qualitative level, accuracy requirements are low. On the other hand, absolute quantities such as the local magnitude of flow velocity, temperature, and other transport variables require the highest level of accuracy. The validation of model-predicted absolute quantities requires quantification of the prediction errors. This can be accomplished by comparing the CFD solution with experimental data or against highly accurate numerical solutions. 

For the postharvest period, airflow prediction capabilities of CFD models are compared against velocity and temperature measurement data taken from different spatial locations [15,16,17,47,48], measured the local temperature and airflow velocity on sampling points. In addition to the properties of the cool store atmosphere, the temporal history of fruit core temperatures is used to validate predicted cooling rate and produce temperature distribution during the precooling of pomegranate fruit [13,160].

## 7. Future Prospects for Computational Thermo-Mechanical Analysis in Fruit Cold Chains

Computational science is a rapidly growing field involving the development of models and simulations to understand natural systems. Its application spans many disciplines and is now commonly considered a third mode of scientific methodology, complementing and adding to the traditional experimental and theoretical methodologies [161].

Developments in computer system hardware, firmware, networking, and data management components have increased scientific interest. The advent of the fast Fourier transform (FFT) algorithm, the evolution of the finite volume and finite element methods, the variable step ordinary differential equation solvers, adaptive mesh refinements, B-spline numerical methods, fast matrix algorithms, multigrid techniques, and effective optimization methods figure computational methods as a crucial component of science and engineering. Due to these advancements, the range of solvable problems by computer has increased exponentially. 

Research in computational mathematics increasingly depends on a multidisciplinary approach in which physics and computation are combined as a “computational science”, transcending the usual academic disciplines [161,162]. Successful multidisciplinary teams typically consist of several scientists, engineers, and technicians who together cover the relevant scientific and engineering disciplines, applied mathematics, numerical analysis, statistics, probability theory, computer science, and software engineering. Until recently, high-performance computing (HPC) was largely the preserve of the automotive, aerospace, and financial services industries, but, increasingly, the need for HPC within the life sciences sector has predominated. The advanced computational abilities that are user-friendly, come at a relatively low cost, and offer a robust operational environment provide researchers with access to HPC.

Computational thermo-mechanical analysis in postharvest agri-food sectors, as in many other sectors, is now widely acknowledged as a high-leverage element of the expensive, time-consuming, and difficult experimental methods in preharvest and postharvest activities. The airflow, heat transfer, gaseous, and particulate transport processes during precooling, cold storage, refrigerated transport, and refrigerated display cabinets have been successfully investigated by many researchers. Recently, high-performance computers have enabled the incorporation of detailed geometries and complex mass and heat transport phenomena into the processing system [15,16,17,39]. Now, it is also possible to couple the produce quality model into computational fluid dynamic analysis to quantify and visualize the quality deterioration in time [41]. This was impossible a few years ago.

The CFD method is commonly applied to quantify and visualize the effect of vent holes on the produce cooling rate, cooling uniformity, and accompanying energy usages during the precooling, cold storage, and refrigerated transport of perishables [163,164,165,166,167,168]. Studies in this respect are relatively clear that the available CFD software packages most often have the required model equations to solve the airflow and heat transfer problems. Faithful accounting of geometries of the packaged produce and temperature and velocity boundary conditions are still challenging. Holistic analysis of the thermal performances of cold chain logistics, together with the produce quality and system energy usages, is a recent advance in this area.

Proper design and implementation of a vented package must consider the effect of mechanical forces on the package and on biological tissues. CSD, specifically the finite element method (FEM), is normally used to analyze the relationship between vent hole design (size, proportion, shape, and location) and the strength and mechanical stability of corrugated fiberboard cartons (CFCs), as well as the performances of CFCs in the cushioning and damping of impact, compression, and vibration forces. While FEM is a reliable way to solve static or dynamic problems, there are still challenges due to the complexity of paper material and characterization of temperature and humidity effects on the material properties. Advances are moving towards the incorporation of the stress response of biological tissues into the FEM [169]. 

CFD- and CSD-based cold chain models enable researchers of the postharvest period to perform abstract conceptual design and analysis of alternatives. This approach is creative and novel for performing integrated multi-criteria performance analysis in a more convenient and economical way. This approach reduces the time and cost of experimentation. Nevertheless, CFD/CSD still requires experimental validation before implementation and should be approached carefully. The holistic analysis of the mechanical and thermal performances of ventilated packages, together with the produce quality and system energy, is interesting. We are still at the advent of a true multiscale approach to CFD simulation of postharvest systems, and the first comprehensive studies are to be presented in the coming years. Such a breakthrough will soon be possible with the availability of accurate 3D geometry acquisition tools and correctly implemented turbulence models.

## Figures and Tables

**Table 1:** The most used general-purpose codes in the postharvest computational fluid dynamics application.

Software Package	General Description	Postharvest Applications
COMSOL Multiphysics	Finite-element-based, solves various physics and engineering problems, especially coupled phenomena or multi-physics. Usually used for small uncomplicated systems. Possible to run 2D simulations.	Conversion of ethylene to ethanol during respiration processes occurring inside stored food [49]. Mechanical models of compression and impact on fresh fruits [50]. Modeling the diffusion–adsorption kinetics of 1-methylcyclopropene (1-MCP) in apple fruit and non-target materials in storage rooms [51]. Modeling the downregulation of respiration in pear fruit [52]. Model of gas and moisture exchange [53,54].
ANSYS in Fluent	Finite-volume-based, the most versatile. Solves problems for multiphase flows, chemical reaction, viscous and turbulent, internal and external flows, flow-induced noise predictions, and heat transfer with and without radiation. Possible to run 2D simulations.	Airflow and heat transfer during forced-air precooling of fresh fruit [14,55,56]. Characteristic analysis of humidity control in a fresh-keeping container using CFD model [57]. Analysis of airflow and heat transfer inside fruit-packed refrigerated shipping container [16,17]
ANSYS in CFX	Finite-volume-based. Ansys, Inc. released the new release Ansys 2020 R2 on July 15, 2020. Can only run 3D simulations	Analysis of the spatiotemporal temperature fluctuations inside an apple cool store in response to energy use concerns [15]. Air-assisted orchard spraying [3]. Spatial distribution of gas concentrations in fruit storage containers [58].
Open FOAM	Finite-volume-based numerical algorithms originally developed by the CFD research group at London’s Imperial College in the late 1980s.	Analysis of the effect of ventilated packaging design and cold chain scenarios on the cooling kinetics and fruit quality for each single citrus fruit in an entire pallet [14]. Virtual cold chains [39].

**Table 4:** Objectives, numerical techniques, and results of computational-fluid-dynamics-based analysis in reefer container studies.

Fruit	Numerical Technique	Objectives	Result	Reference
Citrus	FVM ANSYS in Fluent	Investigating a new cold chain protocol.	Ambient loading of fruit in reefer containers for cooling during long-haul marine transport.	[66]
N/A	FVM	Investigating the effect of the azimuth angle on energy consumption.	The introduction of installation of a roof shade at reefer container reduced energy consumption.	[74]
Apple	FVM ANSYS in CFX	Analyzing the air-corrugated carton moisture exchange phenomenon inside reefer containers.	The sealing efficiency was estimated for different situations, with the air circulation devices installed inside, outside, or on both sides of the door.	[21]
Citrus	FVM ANSYS in Fluent	Investigating the impact of cargo stacking methods on temperature distribution.	Non-uniformity of temperature distributions increases with stack height and stack length.	[75]
Table Grapes	FVM ANSYS in Fluent	Investigating the differences among refrigerated trailer models.	Reports significant differences among three airflow design models of refrigerated cargo systems.	[76]
Apple	FVM	Redesigning internal structure for improved cooling rate and uniformity.	Airflow velocity was improved by 5.24–425.04% and the cooling rate improved by 34.0% to 59.5%.	[77]
N/A	NA	Investigating the effect of inlet velocity variation on the cooling speed inside a refrigerated container.	The cooling speed difference between low-speed (4 m/s) and high-speed (10 m/s) fans was about 12 min.	[74,78,79]
Banana	FEM in COMSOL Multiphysics software	Evaluating airflow pattern in reefer container loaded with bananas.	Simulation results predicted the location of the hot spots. Moreover, it was found that the cooling distribution was improved by modification of the scheme for placing pallets in the container, the so-called chimney layout.	[80]
Apple	FVM ANSYS in Fluent	Investigating the cooling rate and cooling uniformity performances of commonly used ventilated packaging boxes.	The study demonstrated the significance of vent holes on the bottom face of packaging boxes and the potential energy-saving opportunities in refrigerated transport systems.	[16,17]

**Table 5:** Examples of different studies on compression strength of CFCs.

Problem	Approach	Software Package	Key Outputs	References
To estimate the static top-to-bottom compressive strength of corrugated packaging with different ventilation openings and holes.	Analytical-numerical approach validated with experimental data	Not applicable	For different hole sizes or locations in no-flap and flap boxes, the estimation error may be reduced up to three times, compared to the simple analytical approach.	[104]
To determine the influence of paperboard carton design on its bulging performance when under compression.	Experimental analysis	Not applicable	To a certain extent, regular slotted cartons with a 5-down footprint had higher compression bulge displacement on the short face with increasing carton height at ambient conditions.	[105]
To analyze the deformation and compressive strength of cartons with different indentation shapes.	Simulation and experimental methods	ANSYS Mechanical	Rhombus indentation and cross-indentation had the greatest influence on the compressive strength of cartons.	[106]
To investigate the strength of corrugated cardboard boxes.	Experimental analysis	Not applicable	As the paper weight increased, the strength of corrugated cardboard increased.	[107]
To propose an analytical model to deduce the compression force for corrugated paperboard packaging.	Mathematical model and experimental method	Not applicable	Analytical results compared with experimental showed a good correlation.	[108]
To determine the compression strength of different corrugated fiberboard boxes.	Nonlinear finite element analysis that considers geometric nonlinearity and material nonlinearity validated with experimental methods	MSC Marc	Boxes with a hand hole in their end panel had less compression strength than those without holes.	[109]
To investigate the effect of carton configuration on compression strength.	Nonlinear finite element analysis and experimental methods	MSC Marc	The compression strength of double-walled corrugated cartons (BC-flute) was higher than single-walled cartons (B- and C-flute).	[110]
To evaluate the compression strength of corrugated cartons at storage conditions.	Experimental analysis	Not applicable	The compression strength of the carton decreased with an increase in moisture content.	[23]
To study the effect of multiple creasing lines on the compression strength of corrugated cartons.	Experimental analysis	Not applicable	The carton compression strength decreased significantly due to additional creasing line(s).	[93]
To determine the effects of storage temperature on the moisture content and compression strength of two carton designs.	Experimental analysis	Not applicable	The compression strength of the “Supervent” carton with 34% more vent area was significantly lower than that of the “Standard” carton.	[88]
To study the effects of squareness on the compression strength of corrugated cartons.	Experimental analysis	Not applicable	Square cartons of any flute type performed better than rectangular cartons of the same perimeter and same materials.	[25]
To evaluate the effects of geometric parameters on carton compression strength.	Simulation and experimental methods	MSC Nastran	Vent number, area, orientation, and shape affected the compression strength of the carton. Rectangular vent holes better retained the carton strength.	[86]
To analyze possible deformation shapes of corrugated paperboard cartons under compression.	Experimental analysis	Not applicable	Varying skews with side walls bending in both convex and concave directions were observed during BCT.	[111]
To assess the effect of aspect ratio on corrugated carton compression strength.	Experimental analysis	Not applicable	The compression strength reached the maximum when the aspect ratio at 1.6.	[112]

**Table 6:** Examples of some conditions used for simulated vibration of packed fresh fruit.

Produce	Frequency (Hz)	Acceleration (g)	Duration (min)	Packaging Methods Used	Damage and Quality Evaluation	References
Fresh Fig	3	0.06	30	Polystyrene packaging boxes	Yes	[133]
	16	0.26	30	Cardboard boxes		
Watermelon	7.5, 13	0.3, 0.7	30, 60	Corrugated containers	Yes	[134]
Kiwifruit	7.5, 13	0.3, 0.7	N/A	Wooden bin	Yes	[135]
Kiwifruit	20	0.9	300	Foam-rubber cushion	Yes	[136]
Banana	3.5	0.1	120	Styrofoam sheets	Yes	[137]
				Corrugated fiberboard boxes		
Banana	PSD spectra	0.36	180	Corrugated cartons	Yes	[119]
Peaches	N/A	0.56	90	Polyurethane	Yes	[138]
				Corrugated fiberboard boxes		
				Expandable polyethylene		
Apricot	17, 20	0.7	15, 30	Reusable plastic containers	Yes	[139]
Apple	9, 12, 15	0.9	240	Corrugated paperboard cartons	Yes	[131]
Apple	7.5, 10 13	0.3, 0.5, 0.7	N/A	N/A	Yes	[140]
Strawberry	9	N/A	2880	Polyvinyl Chloride (PVC) containers	Yes	[141]
				Crates made of cardboard		
Strawberry	3	0.4	0.83, 2.5	Polyethylene terephthalate (PET) vented punnets	Yes	[142]
	4	0.8				
	5	1.1				

## Data Availability

Not applicable.
